# Correlation Between Sun Protection Factor and Hair Color Difference Index in a New Method for Evaluating Sunscreens’ Ultraviolet Protection Efficacy in Hair: An Experimental Study

**DOI:** 10.1111/srt.70299

**Published:** 2025-12-17

**Authors:** Jee‐Hye Oh, Go‐Woon Choi, Sung‐Ha Park, Byoung‐Jun Park

**Affiliations:** ^1^ Kolmar Korea Co., Ltd. Seoul Republic of Korea

**Keywords:** evaluating UV protection, hair, hair color difference, suncare, sunscreen, UV

## Abstract

**Background:**

This study aimed to establish a novel method for evaluating ultraviolet (UV) protection efficacy on hair, focusing on the relationship between sun protection factor (SPF) and UV‐induced hair color changes. We propose a hair SPF (HSPF) that measures the extent of hair color change following UV irradiation.

**Materials and Methods:**

In in vitro tests, we used UV standard samples with known SPF values (P2, P5, and P8) to construct a standard curve according to hair color changes before and after UV irradiation. Hair samples were analyzed using the CIELAB color space system, calculating the Δ*E* value to represent the color difference. To assess the UV protection properties of various hair care formulations (oils, essences, and balms), we compared their HSPF with the standard curve. Meanwhile, in vivo tests followed the ISO 24444 guidelines, involving ten participants to validate the correlation between the in vitro and in vivo methods.

**Results:**

Higher SPF products resulted in smaller ΔE values, indicating less color fading and greater protection. The in vitro HSPF values strongly correlated with the in vivo SPF results, particularly for oils and balms (*R* ^2^ = 0.734). However, formulations with high‐moisture content exhibited lower correlation.

**Conclusion:**

The HSPF offers a new perspective on the level of hair protection from UV damage and may be promising for the future development and efficacy testing of UV protection products for hair.

## 1 Introduction

Exposure to sunlight on the skin is inevitable, and skin damage caused by excessive ultraviolet (UV) rays has been a public health problem. UV rays are categorized by their wavelength: UVC (with short wavelengths, 200–280 nm); UVB (with medium wavelengths, 280–320 nm); and UVA (with long wavelengths, 320–400 nm). In particular, UVC is mostly absorbed and scattered by the ozone layer, thereby having less direct effect on the skin than UVA and UVB [1, 2]. Although UV rays provide benefits, such as synthesizing vitamin D and treating psoriasis and vitiligo [3, 4, 5, 6], excessive exposure causes oxidative stress on the skin, leading to photoaging, sunburn, and skin cancer [7, 8, 9].

UV rays adversely affect not only the skin but also the hair. Hair consists of three main layers: the outer cuticle, inner cortex, and medulla [10, 11]. The color of hair is determined by the type and distribution of melanin granules within the cortex [12].

UVA penetrates deep into the cortex, promoting reactive oxygen species (ROS) production, while UVB reaches approximately 5 µm into the hair, affecting the absorption of major protein chromophores, breaking down melanin pigments, disrupting cysteine disulfide bonds in keratin, and causing structural damage to the cuticle [13]. As a result, UV radiation not only degrades key molecular components of hair, such as keratin proteins and melanin, but also leads to dryness. Long‐term exposure can further contribute to vitamin D deficiency and hair color changes [14, 15, 16, 17].

UV wavelengths within 245–400 nm can cause hair damage; they affect the proteins in the cuticles and cortex, cell membrane complexes, and melanocytes, resulting in hair color changes [18]. Given these harmful effects, sunscreen application is becoming more common to prevent short‐ and long‐term damage to the skin and hair.

The sun protection factor (SPF) measures how effective a sunscreen can protect the skin from UVB rays. SPF is calculated by comparing the minimum erythema dose (MED) on sunscreen‐applied skin with that on nonapplied skin. Each country conducts SPF testing through standardized in vivo tests [19, 20].

However, unlike the skin wherein in vivo testing for sunscreens provides a protective index such as SPF, no sufficient method is available for evaluating sunscreen efficacy on hair. Skin protection can be immediately assessed through sunscreens and their response to UV rays, but hair has a fundamentally different structure, making it unsuitable for evaluation through skin‐based methods. Compared with the extensive research and methods for evaluating sunscreen efficacy on skin, research on hair is relatively lacking.

Therefore, this study aimed to develop an evaluation method that estimates the effect of UV rays on hair, and to assess its correlation with the ISO 24444 evaluation method, which evaluates sunscreens’ UV protection efficacy on skin, published by the International Organization for Standardization. By confirming hair color changes caused by UV irradiation and applying UV standards with varying protective effects, the hair SPF (HSPF) evaluation method was established. This newly developed method is expected to be significantly useful in developing UV protection products and evaluating their efficacy on hair.

## 2 Materials and Methods

### 2.1 Hair Sample Preparation

Black virgin hair with no history of chemical treatment was purchased from Hanall Enterprise Co., Ltd. (Republic of Korea) to minimize external variables related to individual hair damage and to ensure reliable comparison with the in vivo test. The hair samples were combed and adjusted to an appropriate width (approximately 4–5 cm). To assess the effect of UV irradiation, we first pretreated the samples with fluorescence staining using Dansyl chloride solution (10% in Acetone; TCI America, USA). This solution was diluted 50 times to a final concentration by diluting it five times with acetone. The dyeing solution was then applied to the prepared hair and allowed to react for 30 min under light‐blocking conditions at room temperature.

After the reaction, we thoroughly washed out the residual solution using distilled water until the solution became clear, ensuring the removal of any residual dye. Then, the hair samples were dried for at least 10 h under constant temperature and humidity conditions, protected from light. Once dried, the hair was arranged to prevent scattering and securely fixed to a black plate that was unaffected by UV rays.

### 2.2 Hair Color Measurement (L*a*b*)

**FIGURE 1:**
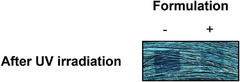
Effect of UV filter formulation on hair L*a*b* distribution under UV exposure. Fluorescently dyed hair samples were divided into two sections: one treated with a formulation containing a UV filter (right) and the other left untreated (left). Both sections were exposed to an identical amount of UV irradiation. As shown, the application of a UV filter‐containing formulation resulted in noticeably less color change in the treated area, which corresponds to a distinct difference in Lab values, reflecting the formulation's UV protection efficacy.

The UV protection efficacy of each formulation was evaluated using fluorescently dyed hair samples, which were prepared and imaged using the Mark‐Vu device (PSI Plus, Republic of Korea). This system enables high‐resolution imaging under various lighting conditions, including both visible and ultraviolet (UV) light. Images were captured before and after UV exposure to extract Lab color coordinates, and quantitative colorimetric analysis was conducted using ImageJ software. The resulting values were used to calculate the color difference (Δ*E*), which served as the metric for assessing the degree of photoprotection provided by each formulation. A representative image of the experimental setup and the visual differences in hair color due to UV protection is shown in Figure 1.

### 2.3 Sunscreen Samples/Formulation

For the evaluation samples, we selected three classifications of commonly used hair products, namely, oil, essence, and balm. Three types of hair essences and oils were developed by Kolmar Korea, and the balm formulation was evaluated using a purchased prototype. In all formulations developed by Kolmar Korea, each UV filter was incorporated at a standardized concentration of 0.45% (w/w). Depending on the formulation type, between four and nine different UV filters were included, resulting in total UV‐filter concentrations ranging from approximately 1.8% to 4.05%. This standardized concentration was chosen in accordance with Korean cosmetic regulations, which restrict higher levels of UV filters to approved sunscreen products. The detailed composition of UV‐protective ingredients in each formulation is provided in Table 1.

**TABLE 1:** Contents of test products.

No.	Product	UV filter	UV absorber
1	Essence A (KEA)	‐ Polysilicone‐15 ‐ Ethylhexyl triazone ‐ Bis‐ethylhexyloxypheno ‐ Methoxyphenyl triazine	Organic
2	Essence B (KEB)	‐ Polysilicone‐15 ‐ Ethylhexyl triazone ‐ Bis‐ethylhexyloxypheno ‐ Methoxyphenyl triazine	Organic
3	Oil (KO)	‐ Polysilicone‐15 ‐ Ethylhexyl triazone ‐ Bis‐Ethylhexyloxypheno ‐ Methoxyphenyl triazine ‐ Diethylhexyl butamido triazone ‐ Ethylhexyl salicylate ‐ Homosalate ‐ Ethylhexyl methoxycinnamate ‐ Diethylamino hydroxybenzoyl hexyl benzoate	Organic
4	Balm (EB)	‐ Ethylhexyl methoxycinnamate ‐ Diethylamino hydroxybenzoyl hexyl benzoate ‐ Bis‐ethylhexyloxyphenol methoxyphenyl triazine	Organic

Abbreviation: UV, ultraviolet.

### 2.4 Sunscreen Application

Sunscreen was applied to a polymethylmethacrylate (PMMA) plate. The UV‐transmitting PMMA plate, with a rough surface and a minimum area of 16 cm^2^, was purchased from Helioscreen (Helioscreen, Belgium). Sunscreen was applied to PMMA plates at a thickness of 1.3 mg/cm^2^ and allowed to dry for at least 15 min under constant temperature and humidity conditions (22 ± 2°C, relative humidity 40%–60%), identical to those used in in vivo test. After stabilization, we placed the sunscreen‐coated plate over the hair samples and applied UV rays to confirm the sunscreen's protective effect against UV rays on the hair.

### 2.5 Hair UV Irradiation

For UV irradiation, we used 601 Multiport Solar Simulator (Solarlight Co., USA). This device can stably and uniformly irradiate both UV‐A and UV‐B light, emitting light of various wavelengths simultaneously from six output ports. The emission spectrum of solar simulated radiation (SSR) is shown in the supplementary figure. To confirm hair color changes caused by UV effects, we set the light intensity of all ports to 800 µW/cm^2^ to ensure consistent irradiation across all areas.

### 2.6 Standard Curve (HSPF Curve) Construction

We constructed a standard curve to establish the correlation between sunscreen's UV protection efficacy and hair color difference. We used the standard samples P2, P5, and P8 proposed by ISO (Cosmetech Laboratories Inc., USA) for constructing the standard curve. Table 2 shows the UVB protection index of each standard sample.

**TABLE 2:** UVB protection index of SPF reference standard.

Product	Mean SPF	Acceptance limits	
		Lower limit	Upper limit
P2	16.1	13.7	18.5
P5	30.6	23.7	37.4
P8	63.1	43.9	82.3

Abbreviation: SPF, sun protection factor.

Sunscreen application and UV irradiation were performed as described in Sections 2.3 and 2.4, respectively. The hair samples’ L*a*b* values were confirmed through image analysis before and after UV irradiation, and the △*E* value was calculated according to the CIELAB color space formula. The calculated △*E* value correlated with the UV protection index (SPF), and the HSPF in vitro standard curve equation was derived from this data.

### 2.7 In Vitro/In Vivo Correlation Verification Using Hair Products

The in vivo UV protection index was evaluated to determine the correlation between the following two methods: the in vitro method, which evaluates the UV protection efficacy of newly developed hair products, and the generally accepted in vivo UV protection evaluation method.

The in vitro evaluation method determined the hair light protection index by using the △*E* value before and after UV irradiation, as described in Sections 2.3 and 2.4. This method was done by confirming the correlation between △*E* and the UV protection index through the constructed standard curve.

Equation 1. Calculation of individual typology angle (ITA)

(1) $$\mathrm{ITA}=\left\{\mathrm{arctangent}\frac{\left(L*\mathrm{value}\;-50\right)}{b*\;\mathrm{value}}\right\}\frac{180}{\pi }$$

The in vivo evaluation was conducted according to ISO 24444:2019 and approved by the Institutional Review Board of Kolmar Korea (IRB no. 2024‐01‐008) before the study. We recruited ten participants according to the inclusion and exclusion criteria outlined in the lSO guideline [19]. The test area, which was a clean, dry back without skin damage, was divided into flat sections, excluding the spine. The skin color of each participant was measured using a chromometer. The suitability of each participant was determined by calculating the value using Equation (1). A value of at least 28° with a variation of below five between test areas was deemed acceptable. Additionally, the average ITA° of the 10 participants had to be within 41°–55°, with at least one participant falling into each of the following ranges: 28°–40°, 41°–55°, and >56°.

During the in vivo evaluation, we used the same hair products as in the in vitro assessment, under identical environmental conditions (22 ± 2°C, 40%–60% relative humidity). For the control, we chose one standard product according to the expected UV protection index. We used a rubber thimble to uniformly apply the product to the divided test areas at 2 mg/cm^2^ quantity. The applied products were then stabilized for 15–30 min, followed by UV irradiation.

For the light source, we utilized the Multiport Solar Simulator, 601–300 W (Solar Light, USA0), which has a continuous radiation spectrum similar to sunlight. This device has a 300 W xenon arc lamp that does not exhibit a specific peak as a light source. Wavelengths below 290 nm were removed by a WG320 filter, and those above 400 nm in the visible light region were removed by a UG11 filter. Consequently, an artificial light source with wavelengths of 290–400 nm, which is suitable for the experiment, was provided. UV rays emitted from the lamp were delivered in a circular shape with approximately 0.8 cm diameter through six ports using a dichroic mirror and collimating lens. The light intensity for each port was set to ensure that the final irradiated light amount was approximately 15% (Table 3).

**TABLE 3:** Intensity and quantity of light guide.

Light guide	1	2	3	4	5	6
Intensity of light (μ w/cm^2^)	368	424	487	560	644	742
Ratio (%)	15					

The UV protection index (SPF) of each sample was calculated using Equation (2). The MED was determined by at least two people according to the grading system shown in Table 4 for objective evaluation. If a discrepancy occurred in their judgments, the lower MED was adopted.

**TABLE 4:** Grading scale for ultraviolet‐exposed test subsites.

Grade	Contents
0	No erythema present;
0.5	Ambiguous erythema, and/or no clear border, and/or not filling more than 50 % of the exposure subsite;
1	Perceptible unambiguous erythema with defined borders filling more than 50 % of the exposure subsite (MED if it is the lowest exposure dose with grade 1)
2	Moderate to intense erythema.

Equation 2. Calculation of SPF

(2) $$\mathrm{SPF}=\frac{\mathrm{MED}\;\mathrm{on}\;\mathrm{product}\;\mathrm{protected}\;\mathrm{skin}\;\left(\mathrm{MEDp}\right)}{\mathrm{MED}\;\mathrm{on}\;\mathrm{unprotected}\;\mathrm{skin}\;\left(\mathrm{MEDu}\right)}\;\;$$

### 2.8 Statistical Analysis

All statistical data were analyzed using GraphPad Prism 5 software. Significant differences at a p‐value less than 0.05 were evaluated using one‐way analysis of variance.

## 3 Results

### 3.1 In Vitro Evaluation of UV Standard Samples

To establish a correlation curve between hair color change and UV protection index, we conducted an in vitro evaluation using ISO‐standard UV reference samples (P2, P5, and P8). The study included four groups: No treat group (control group with no product applied), and three treatment groups corresponding to P2, P5, and P8.

**FIGURE 2:**
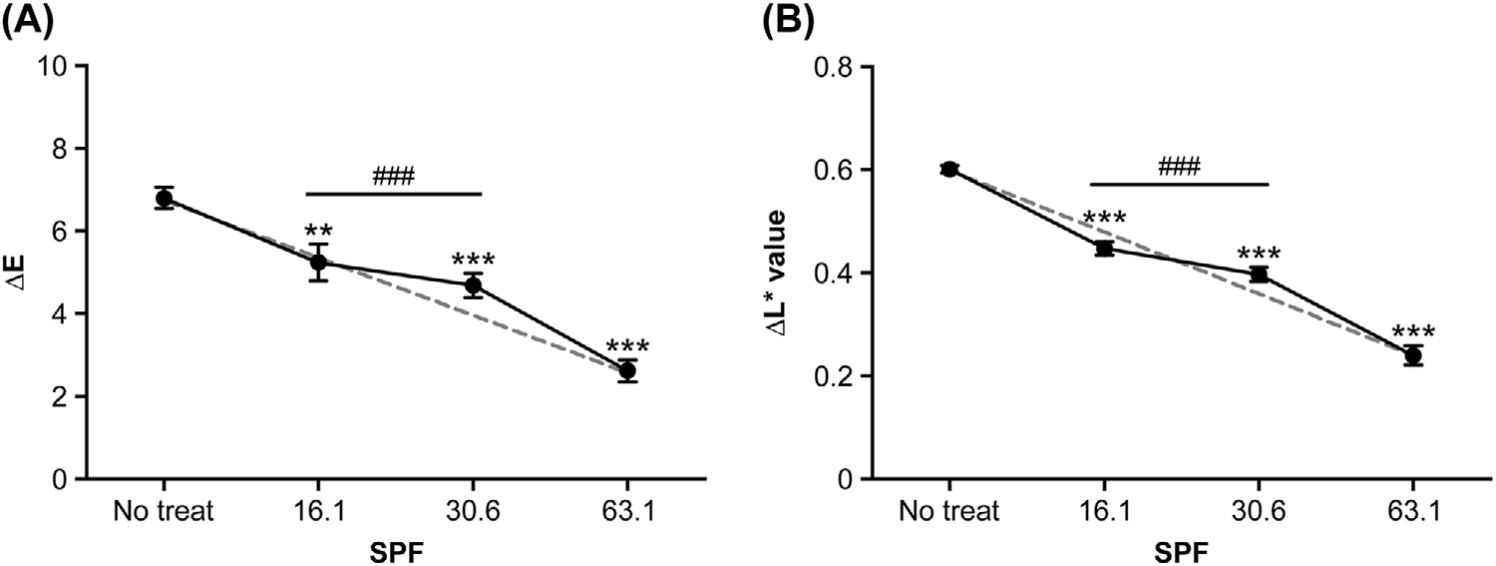
In vitro results of SPF reference standard (P2, P5, and P8). ΔE decreases by sun ‐protection evaluation. The Data represent the means ± SEM, *n* = 20, statistically significant at **p < 0.05, ***p < 0.001 compared to with No‐ treatment group, ###*p* < 0.001 compared with the P8 group. No treat, control group with no product applied; SPF, sun protection factor.

The evaluation of the standard samples with different UV protection indices yielded the following results. The △*E* of P2, P5, and P8 with mean SPF values of 16.1, 30.6, and 63.1 was 5.24 ± 2.02, 4.68 ± 1.30, and 2.62 ± 1.22, respectively (Table 5). Thus, higher UV protection may result in less chromatic difference (△*E*) caused by UV irradiation in the hair, suggesting that the UV protection can effectively prevent hair color change. The HSPF standard curve was constructed according to the results obtained from the standard samples. Figure 2 shows the formula and graph.

**TABLE 5:** In vitro results of the SPF reference standard (P2, P5, and P8).

Product name	SPF mean value	△ Color difference (mean ± SD)	△L* value difference (mean ± SD)
No treat	0	6.80 ± 1.13	0.60 ± 0.03
P2	16.1	5.24 ± 2.02	0.45 ± 0.06
P5	30.6	4.68 ± 1.30	0.40 ± 0.07
P8	63.1	2.62 ± 1.22	0.24 ± 0.09

Abbreviations: No treat, control group with no product applied; SD, standard deviation; SPF, sun protection factor.

### 3.2 HSPF In Vitro Evaluation of Hair Care Formulations Using Standard Curves

**FIGURE 3:**
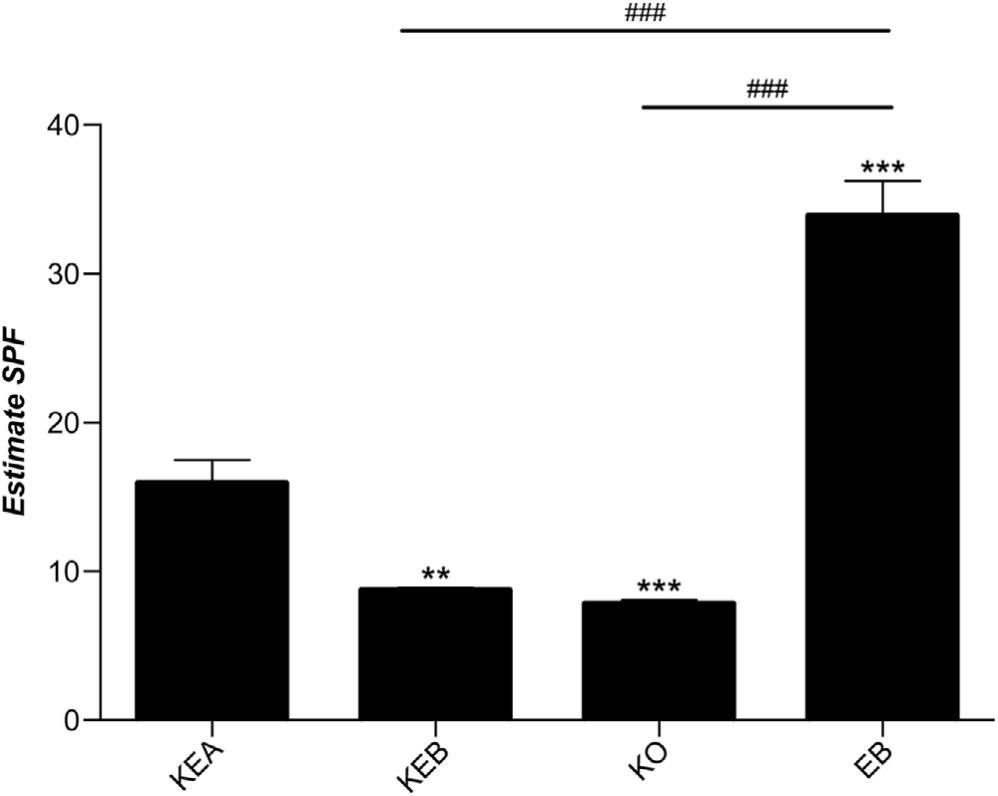
HSPF in vitro results of the test products. KEA, essence A; KEB, essence B; KO, hair oil; EB, hair balm; SPF, sun protection factor. Data represent the means ± SEM, *n* = 20, statistically significant at **p < 0.05, ***p < 0.001 compared with the KEA group, ###*p* < 0.001 compared with the EB group.

An in vitro SPF evaluation was conducted to predict the hair light protection index of various hair care formulations using the HSPF standard curve constructed from the standard samples with verified UV protection indices. After confirming the L*a*b* values through image analysis before and after UV irradiation in each formulation, we calculated the △*E* value using the CIELAB color space formula. When these values were substituted into the HSPF curve, the estimated SPFs were as follows: essence A (KEA), 15.9 ± 6.9; essence B (KEB), 8.8 ± 0.6; oil (KO), 7.8 ± 1.0; and balm (EB), 33.9 ± 5.4 (Figure 3).

### 3.3 In Vivo Evaluation of Hair Care Formulations (ISO Method)

To confirm the correlation between the in vitro hair light protection index and the in vivo sunscreen index derived from the HSPF standard curve, we set the expected SPF according to the in vitro results shown in Section 3.2. The test was conducted according to ISO 24444 (Cosmetics–Sun Protection Test Method—In vivo Determination of the Sun Protection Factor). A total of 10 subjects—nine women and one man—participated in the test, with an average age of 42.9 years (ranging from 25 to 63 years). The mean ITA° was 36.5, with a standard deviation (SD) of 7.02.

KEA, KEB, KO, and EB had SPF values of 5.3 ± 0.5, 7.9 ± 0.9, 5.6 ± 1.2, and 28.5 ± 6.1, respectively (Table 6). The standard samples P2 and P5 met the recognition range (10.7–14.7) of the ISO test method, and the validity of the test results was confirmed, given that the 95% confidence interval (CI) value of the evaluation sample was 17% or less (Table 7).

**TABLE 6:** In vivo SPF results of the test products.

SPF		KEA	KEB	KO	EB
Test product	HSPF in vitro (mean integer value)	15	8	7	33
	In vivo results	5.3 ± 0.5	7.9 ± 0.9	5.6 ± 1.2	28.5 ± 6.1
Standard product	P2	15.8 ± 4.6	17.4 ± 3.1	16.7 ± 6.2	17.9 ± 4.7
	P5	31.1 ± 9.8	28.4 ± 9.9	29.0 ± 9.8	29.8 ± 10.9

Abbreviations: EB, hair balm; HSPF, hair sun protection factor; KEA, essence A; KEB, essence B; KO, hair oil; SPF, sun protection factor.

**TABLE 7:** Validation of the in vivo results.

Product name	mean SPF	*s*	*n*	*t*	*c*	CI	95% CI	17% of mean SPF	CI[%] ≤ 17%
KEA	5.3	0.5	10	2.262	0.3	5.7	4.99–5.59	0.90	Proven
P2	15.8	4.6	Acceptance limit 13.7–18.5						
P5	31.1	9.8	Acceptance limit 23.7–37.4						
KEB	7.9	0.9	10	2.262	0.6	7.6	7.32–8.52	1.35	Proven
P2	17.4	3.1	Acceptance limit 13.7–18.5						
P5	28.4	9.9	Acceptance limit 23.7–37.4						
KO	5.6	1.2	10	2.262	0.9	16.2	4.56–6.46	0.95	Proven
P2	16.7	6.2	Acceptance limit 13.7–18.5						
P5	29	9.8	Acceptance limit 23.7–37.4						
EB	28.5	6.1	10	2.262	4.4	15.4	24.1–32.9	4.85	Proven
P2	17.9	4.7	Acceptance limit 13.7–18.5						
P5	29.8	10.9	Acceptance limit 23.7–37.4						

Abbreviations: CI, confidence interval; EB, hair balm; KEA, essence A; KEB, essence B; KO, hair oil; SPF, sun protection factor.

### 3.4 Validation of the Correlation between HSPF In Vitro and ISO In Vivo

The correlation between the developed in vitro HSPF evaluation results and the human application test results shown in Section 3.3 was analyzed using Pearson's correlation coefficient method. We compared the correlation between the in vitro and in vivo test methods for each product. KEA's in vitro and in vivo tests showed a low correlation (*R* ^2^ = 0.105). Conversely, the in vitro and in vivo methods for KEB, KO, and EB demonstrated high correlations (*R* ^2^ = 0.553, 0.509, and 0.734, respectively) (Table 8). Therefore, for KEB, KO, and EB formulations, the HSPF in vitro evaluation method significantly correlated with the existing ISO in vivo evaluation method, a method for evaluating sunscreens’ UV protection efficacy for humans.

**TABLE 8:** Correlation between the in vitro HSPF and in vivo ISO SPF methods.

Product name	HSPF in vitro (using △*E*, *n* = 20)	SPF in vivo (*n* = 10)	$R^{2}$	HSPF in vitro vs. ISO SPF in vivo
KEA	15.9 ± 6.9	5.3 ± 0.5	0.11	Poor correlation
KEB	8.8 ± 0.6	7.9 ± 0.9	0.55	Good correlation
KO	7.8 ± 1.0	5.6 ± 1.2	0.51	
EB	33.9 ± 5.4	28.5 ± 6.1	0.73	

Abbreviations: HSPF, hair sun protection factor; EB, hair balm; KEA, essence A; KEB, essence B; KO, hair oil; SPF, sun protection factor.

## 4 Discussion

This study is the first to link two main factors to measure the UV protection efficacy of different sunscreen formulations according to the UV protection index, specifically the hair UV protection index (HSPF), before and after UV irradiation. While many studies have reported on hair bleaching caused by the destruction of melanin pigments in the hair cortex from UV exposure [14], only few have examined the extent of hair color changes according to the UV protection index (SPF) or the methods of evaluating the UV protection efficacy of hair products. Therefore, this study established and tested the following hypotheses according to the correlation between UV protection efficacy and hair color retention: UV radiation amount is associated with hair bleaching.

Given the same amount of light, the amount of light penetrating the hair will be reduced depending on the SPF. The application of formulations containing UV filters showed in distinct L*a*b* color distributions. If a product with high UV protection is applied, the hair will fade less after UV irradiation compared with before. In other words, SPF, which indicates UV protection, is related to hair color difference. By understanding this relationship, the UV protection effect of a product on hair can be possibly determined through the color difference before and after UV irradiation.

To test these hypotheses, we conducted an in vitro evaluation using standard UV clinical samples.

Dansyl chloride exhibits different fluorescence reactions under ultraviolet light depending on the degree of hair damage [21]. To ensure accuracy, we used intact virgin hair, and the results confirmed that a higher UV protection index effectively reduces the amount of light penetrating the hair through the PMMA plate. Consequently, this leads to smaller differences in hair bleaching compared to samples with a lower UV protection index (Figure 2). This finding aligns with previous studies showing that higher UV exposure leads to more severe hair bleaching [22, 23]. Through these results, we have established the correlation between hair color differences before and after UV irradiation and the sunscreen's blocking effect (SPF) using the standard samples.

**FIGURE 4:**
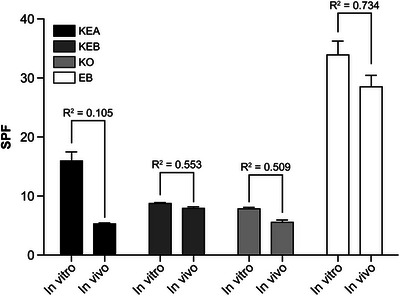
Correlation of the in vitro HSPF and in vivo ISO SPF methods. KEA, essence A; KEB, essence B; KO, hair oil; EB, hair balm; SPF, sun protection factor.

Hypothesis 3 was confirmed through in vitro and in vivo evaluations of various hair care products with unknown SPF. The HSPF value was calculated using the in vitro HSPF evaluation method, which we have developed. An in vivo double‐check using the ISO SPF index evaluation method confirmed a high correlation between the two methods for all sunscreen formulations, except KEA (Figure 4).

The issue of deviation between the in vitro evaluation method using PMMA plates and the in vivo evaluation method remains challenging. Depending on the formulation, sunscreen shows different spreading patterns when applied, and the characteristics of the human skin and plate application area can affect the in vitro/in vivo correlation. Various factors, such as the spectrum of transmission, the amount of the applied product, the process of application, the proficiency of the applicator, and the roughness of the plate can influence in vitro evaluation [21, 22, 23, 24, 25, 26].

In vitro evaluation may also be affected by interfacial tension from the adherence of the formulation during spreading. S. Marguerie evaluated the in vitro/in vivo correlation of two formulations with different cohesiveness using the same PMMA plate and found that O/W emulsions with water as the base had low adherence and a poor correlation compared with formulations with high cohesiveness [24].

KEA contains considerably higher moisture than other hair care formulations evaluated. When applied to the PMMA plate, it exhibited low cohesion, resembling water droplets. This characteristic likely contributed to the low correlation between HSPF in vitro and ISO in vivo.

This study is significant because it proposes a new method to measure UV protection efficacy in the hair, which has been difficult to evaluate previously, by establishing a correlation between UV protection efficacy and hair color retention.

However, this research has limitations that need to be addressed. First, this study focuses solely on Asian black hair, errors may arise when evaluating African or European hair. Jae Hong Ji et al. observed morphological changes in hair lipids after UV irradiation across three ethnic groups and found that Asian hair contained higher lipid content than European and African hair. They also reported that Asian hair exhibited less damage after UV exposure compared to the other two hair types [27]. Given that different hair types respond differently to UV radiation, further research is necessary to explore the correlation between HSPF and SPF across a broader range of hair types beyond Asian black hair.

Second, the evaluation was conducted using a PMMA plate, a UV‐transmitting medium, rather than direct application to hair, thereby causing interference. The PMMA plate has low interaction with high‐moisture formulations, such as KEA, making the evaluation of these formulations challenging.

Third, the standard curve was constructed using verified sunscreens, limiting the UV protection indices available. Only five standard SPF samples have been proposed; hence, color difference data for various UV protection indices could not be calculated comprehensively (e.g., SPF 5, 10, 15, 20, 25, and 30).

Nevertheless, this study is meaningful because it suggests a new method to evaluate UV protection efficacy in the hair, an area where such methods have not been developed. By addressing UV‐induced hair color differences according to sunscreen efficacy, our study estimated the relationship between these parameters. Future studies could establish a new gold standard for evaluating the UV protection index in the hair by excluding PMMA plate interference through direct formulation application and enhancing the standard curve with various verified UV protection agents.

## 5 Conclusion

This study demonstrated the feasibility of using HSPF in measuring the changes in hair color caused by UV rays and the effect of UV rays on the hair to determine the UV protection effect of hair products, providing a new tool for product development and efficacy testing in this field. The in vitro HSPF was found to be significantly correlated with the in vivo results obtained through the ISO human body application test method. Thus, this research presents a new method to evaluate the effectiveness of UV protection products for hair, which is generally difficult to evaluate. In addition, this method will be of great help in the sun protection market for hair. However, future studies need to refine the methodology by applying products directly to the hair and expanding the range of standard UV protection indices used in the evaluation.

## Ethics Statement

The study followed the Declaration of Helsinki and was approved by the Korean public Institutional review board of R&D Complex of Kolmar Korea Co, Ltd. (Approval number: 2024‐01‐008). All the participants were fully informed about the study process before obtaining written informed consent.

## Conflicts of Interest

All authors are employees of Kolmar Korea Co., Ltd. The authors report no other potential conflicts of interest in this work.

## Supporting information




**Figure S1** Emission spectra of individual ports under solar simulated radiation (SSR).

## Data Availability

The datasets used and/or analyzed during the current study are available from the corresponding author (Byoung‐Jun Park; A2001; email: A2001@kolmar.co.kr) upon reasonable request.
