# Supplementary figures and images for: Correlation Between Sun Protection Factor and Hair Color Difference Index in a New Method for Evaluating Sunscreens’ Ultraviolet Protection Efficacy in Hair: An Experimental Study
Source: Skin Res Technol. 2025 Dec 17;31(12):e70299. doi: 10.1111/srt.70299 (PMC12712344; doi:10.1111/srt.70299)

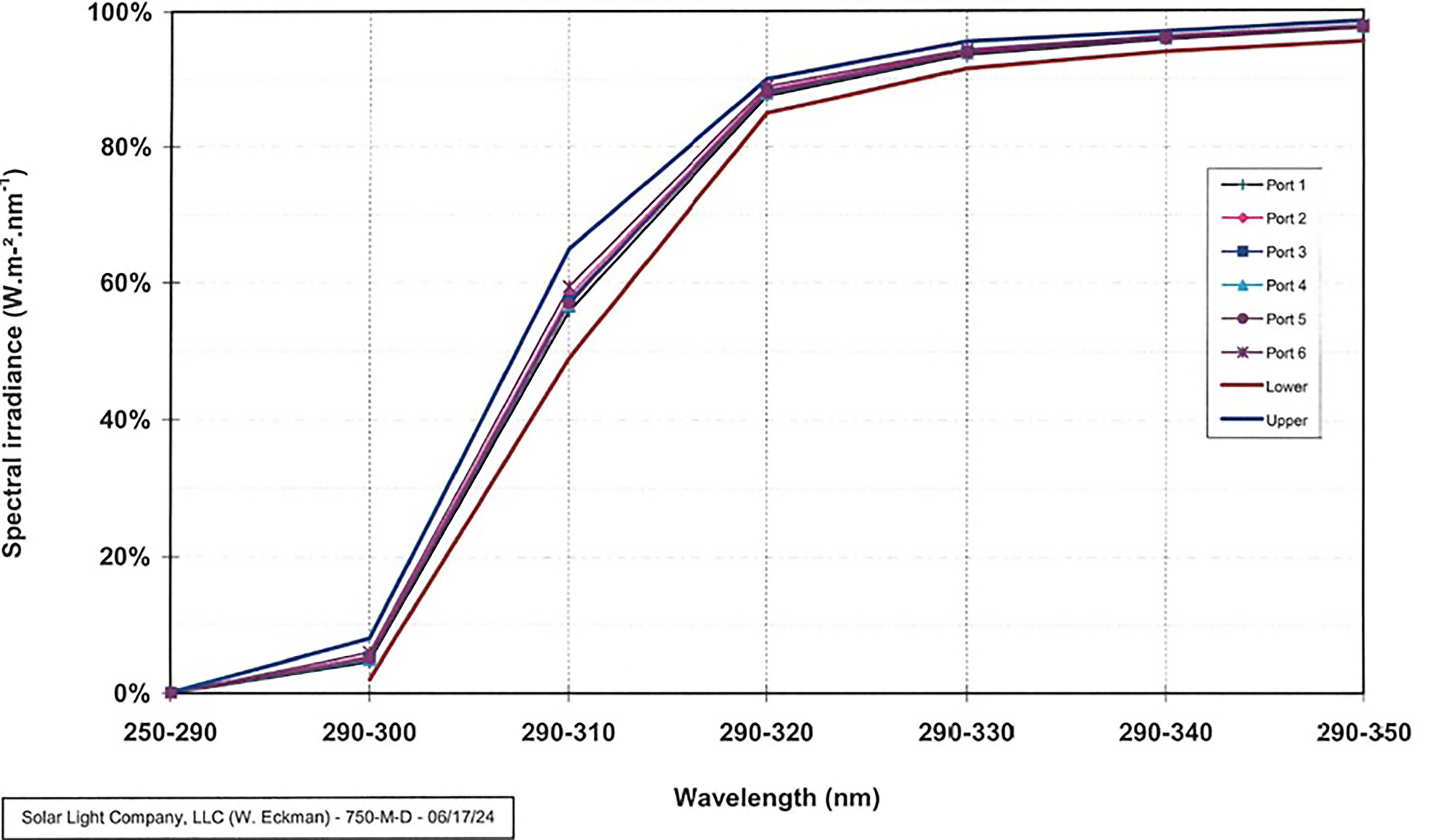

Supplement: Supplementary file 1 — Figure S1 Emission spectra of individual ports under solar simulated radiation (SSR). [file SRT-31-e70299-s001.jpg]
